# Body mass index, physical activity, and severe periodontitis– a cross-sectional HUNT4 Oral Health Study

**DOI:** 10.1186/s12903-026-07882-x

**Published:** 2026-02-18

**Authors:** Mariam Reda, Odd Carsten Koldsland, Laxmi Bhatta, Abhijit Sen

**Affiliations:** 1 Oral Health Centre of Expertise in Western Norway, Bergen, Norway; 2 Department of Periodontology, Institute of Clinical Dentistry, Faculty of Dentistry, University of Oslo, Oslo, Norway; 3 HUNT Center for Molecular and Clinical Epidemiology, Department of Public Health and Nursing, Norwegian University of Science and Technology, Trondheim, Norway; 4 FIU-PH, Division of Mental Health Care, St Olav’s Hospital, Trondheim, Norway; 5 Center for Oral Health Services and Research (TkMidt), Trondheim, Norway; 6 Department of Public Health and Nursing, Faculty of Medicine and Health Sciences, Norwegian University of Science and Technology (NTNU), Trondheim, Norway

**Keywords:** Body mass index, Exercise, Physical Activity, Periodontitis, Oral health

## Abstract

**Background:**

The association between body mass index (BMI), physical activity (PA), and periodontitis remains unclear, particularly how BMI combined with activity levels affects periodontitis. This study aimed to assess the associations between BMI, its combination with PA, and periodontitis severity in Norwegian adults.

**Methods:**

This cross-sectional study included data from participants aged ≥ 20 from the Norwegian HUNT4 Oral Health Survey (2017─2019) (*N* = 4,384). BMI (kg/m^2^) was categorized as normal weight (18.5–24.9 kg/m^2^), overweight (25–29.9 kg/m^2^), and obesity (≥ 30 kg/m^2^). The PA score was constructed based on frequency, duration, and intensity (inactive/low, moderate, high). The combination of BMI and PA was classified into four groups: (1) normal weight, active; (2) normal weight, inactive; (3) with overweight-obesity, active; and (4) with overweight-obesity, inactive. The outcome was periodontitis severity (stages), categorized based on the 2017 Classification of Periodontal and Peri-Implant Diseases and Conditions. Prevalence ratios with 95% confidence intervals were calculated using modified Poisson regression accounting for potential confounders.

**Results:**

Obesity was associated with severe periodontitis, whereas moderate PA showed an inverse association. Individuals with overweight or obesity who were physically inactive had a higher prevalence of severe periodontitis compared to individuals who were normal weight and physi­cally active.

**Conclusions:**

These findings suggest that individuals with obesity and low PA are associated with increased periodontitis severity. Further studies are needed to confirm these associations. This cross-sectional study suggests that physical activity may help mitigate the adverse impact of obesity on periodontitis severity, highlighting the need for longitudinal research and supporting the integration of PA recommendations into oral health management strategies.

**Supplementary Information:**

The online version contains supplementary material available at 10.1186/s12903-026-07882-x.

## Background

Periodontitis, as a chronic inflammatory condition, is characterized by plaque biofilms and sustained by immune system dysregulation, which subsequently leads to host microbiota dysbiosis and may cause various health concerns [1, 2]. The severity of periodontitis is now classified using a multidimensional staging system based on the extent of tissue destruction and the complexity of disease management, as introduced by the 2017 World Workshop on the Classification of Periodontal and Peri-Implant Diseases and Conditions (EFP/AAP 2018 classification) [1, 3].

Obesity is a complex, multifactorial disease and a known risk factor for several non-communicable diseases, including oral diseases [4, 5]. Evidence suggests that obesity induces metabolic and immune dysregulation, resulting in a state of chronic low-grade systemic inflammation [6, 7]. Adipose tissue, particularly excess visceral fat, functions as an active endocrine organ and secretes pro-inflammatory cytokines such as tumor necrosis factor-α and interleukin-6, which may alter immune regulation and amplify inflammatory responses. In the context of periodontitis, where inflammation is initiated by bacterial biofilms, this heightened systemic inflammatory milieu may exacerbate local periodontal inflammation, promote alveolar bone resorption, and impair tissue repair. Obesity-related metabolic disturbances, including insulin resistance and oxidative stress, may further compromise host immune responses and periodontal healing capacity [8]. As a result, obesity may increase an individual’s susceptibility to periodontitis, influence disease progression, and adversely affect treatment outcomes. Consistent with these biological mechanisms, an updated systematic review of observational epidemiological studies has suggested a positive association between Body Mass Index (BMI) and periodontitis [9].

Physical activity (PA) has been demonstrated to protect against many cardiovascular risk factors related to obesity and may further reduce the risk of several non-communicable diseases [10]. Research suggests that higher levels of PA may be associated with a lower risk of periodontitis, potentially due to its anti-inflammatory effects [11]. However, the evidence remains inconclusive, as some studies have reported inconsistent or conflicting findings [12, 13]. This lack of consensus highlights the need for further investigation. Moreover, the potential role of PA in influencing the severity of periodontitis, particularly among individuals with elevated BMI, has not been thoroughly explored.

Several biological pathways have been proposed to explain how obesity could contribute to periodontitis, and how PA could help counteract this effect. PA can enhance insulin sensitivity, thereby lowering the likelihood of developing type 2 diabetes, a well-established risk factor for periodontitis [14]. Another possible mechanism is that PA might reduce visceral fat accumulation and inflammation [15], which could play a significant role in the pathogenesis of periodontitis.

Individuals with high BMI may exhibit varying PA levels, ranging from active to inactive. Those with overweight or obesity who are physically inactive likely represent the most sedentary group. We hypothesize that the combination of high BMI and low PA exerts a greater impact on the prevalence of periodontitis than either factor alone. To date, no studies have yet investigated the association between the combined effect of BMI and PA and periodontitis in adults. Therefore, this study aimed to investigate the cross-sectional associations between BMI, PA, and periodontitis severity in a general Norwegian adult population. Further, we explored the association between the combined effect of BMI and PA and periodontitis.

## Methods

### Study population and settings

**Fig. 1:**
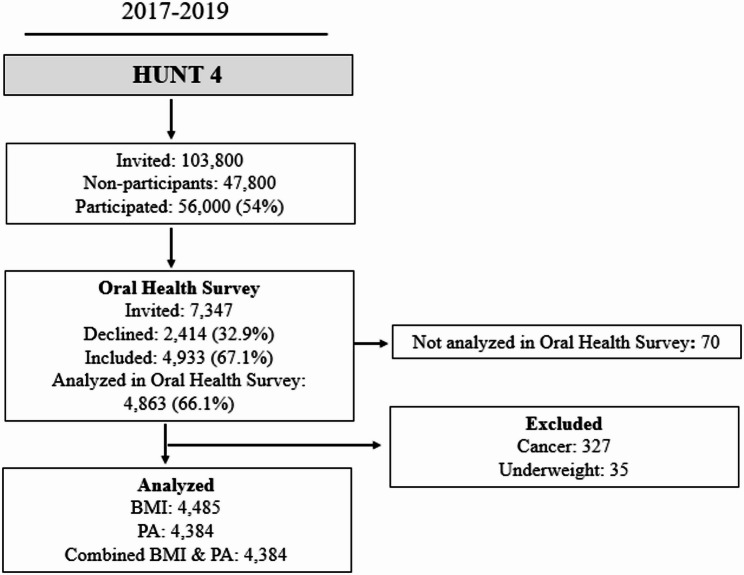
Flowchart of the study participants. PA: Physical activity, BMI: Body mass index

The Trøndelag Health Study (HUNT) is a large population-based study from northern Trøndelag County, Norway [16]. The HUNT study was conducted in four phases: HUNT1, HUNT2, HUNT3, and HUNT4, in the periods 1984–1986, 1995–1997, 2006–2008, and 2017–2019, respectively. During the 2017–2019 period, self-reported questionnaires on the history of chronic illnesses, sociodemographic, behavioral, and lifestyle factors, as well as systolic and diastolic blood pressure measurements and anthropometric measures (weight, height, hip, and waist circumference), were collected at HUNT4 [17, 18]. Furthermore, the Oral Health Survey HUNT4 sub-study was conducted with the objectives of evaluating oral health behaviors and dental conditions/diseases through clinical evaluation (*n* = 4,933) and administering self-reported dental health questionnaires [19]. Individuals aged ≥ 20 years with complete self-reported data on demographics, lifestyle factors, dietary habits, clinical factors, and clinically and radiographically assessed periodontitis data were included in the analyses (*N* = 4,384). Individuals with a previous history of cancer (*n* = 327) or who are underweight (BMI ≤ 18 kg/m^2^, *n* = 35) were excluded. Fig. 1 presents the flowchart of the study participants. The present cross-sectional study is reported in accordance with the STrengthening the Reporting of OBservational studies in Epidemiology (STROBE) guidelines [18].

### Periodontitis

Periodontitis was assessed based on the EFP/AAP 2018 classification by stages, which depend on the disease severity at presentation and the complexity of disease management [1]. In the HUNT4 Oral Health Study, clinical and radiographic measures of periodontal status were conducted by experienced dentists (*n* = 12) and dental hygienists (*n* = 7) from the Public Dental Service, which is present in the northern area of Trøndelag County. The procedure followed is described in another publication [19]. In brief, among other parameters measured during dental examination performed at the HUNT4 field stations were clinical measures of periodontal parameters such as periodontal probing depths (PPDs), bleeding on probing (BoP), suppuration, and mobility grades 2 and 3. The examinations were conducted using mobile dental equipment and a separate mobile stand examination light [19]. Although a more extensive periodontal examination protocol was initially planned, predefined logistical constraints and limitations on examination time at the HUNT4 field stations precluded inclusion of Plaque Index and Gingival Index in the final examination protocol.

PPDs were assessed using a periodontal probe from the WHO (LM 550BSI Probe WHO ErgoNorm, LM Instruments, Parainen, Finland) and were noted in the following intervals: 0–3.5 mm, > 3.5–5.5 mm, > 5.5–8.5 mm, > 8.5–11.5 mm, > 11.5 mm. Oral health examination at HUNT4 field stations included bitewing (BW) and orthopantomogram (OPG) radiographs. Periodontal bone loss was also annotated on the OPG and BW radiographs. Following the data collection in HUNT4, the annotation was completed by skilled periodontists (*n* = 3) at the Institute of Clinical Dentistry, University of Oslo. The inter-rater reliability for PPD measurements was assessed using the intra-class correlation coefficient (ICC) based on a two-way mixed-effects model with absolute agreement as reported in an earlier publication [19]. The findings demonstrated acceptable to excellent inter-examiner reliability, supporting the robustness of the clinical and radiographic measurements used in the present study.

The criteria and case definitions of periodontitis were based on the EFP/AAP 2018 classification scheme [1, 20]. An individual was considered a “periodontitis case” if a distance between the cementoenamel junction (CEJ) and the alveolar bone crest (AC) of more than 1.5 mm at ≥ 2 non-adjacent teeth was registered. The case was determined from the bitewing radiographs and was regarded as “detectable interproximal bone loss” [19]. If the bitewing radiograph was not performed, the distance between the CEJ and AC was defined from the panoramic radiograph (*n* = 64). For the 22 participants with only BW radiographic examinations available, the percentage of bone loss was estimated based on a study of average root lengths [21].

Only “periodontitis cases” were assessed for periodontitis stages. Permanent teeth were included in the periodontal status classification (3rd molars excluded). Root remains were not included. Despite being documented as existing, implants and primary teeth were not included in the periodontal classification. If periodontitis was thought to be a highly likely reason for a missing tooth, it was noted as “missing due to periodontitis”. Moreover, registering the furcation involvement grade II or III was only done when radiographically obvious [19].

Periodontitis stages were determined based on the radiographic bone loss (RBL) and the teeth deemed lost due to periodontitis. The most affected tooth was used to determine the initial stage. To determine the final periodontitis stage, RBL in OPGs, the number of teeth lost due to periodontitis, furcation involvement (grade ≥ 2), vertical bone loss, bite collapse or drifting, or flaring, and PPD > 5.5 mm (used to detect stage III when RBL met the criteria for stage II). If the clinical criteria were not present, the radiographic assessment was used for the categorization (*n* = 71) [19]. Periodontitis stages are classified into four stages: stage I (initial periodontitis), stage II (moderate periodontitis), stage III (severe periodontitis), where advanced treatment is necessary to avoid tooth loss, and stage IV (advanced periodontitis), leading to loss of masticatory function [20].

### Exposures

BMI, PA levels, and their combined associations are assessed as the main exposures in this study. During the HUNT4 data collection, trained nurses performed standardized measurements of weight and height using an InBody770 device, where participants wore light clothing and no shoes [17]. Height and weight were approximately to the nearest centimeter and half kilogram, respectively. BMI was calculated by dividing the weight (kg) by the square of the height (in meters) and classified according to the WHO guidelines: normal weight (18.5–24.9 kg/m^2^), overweight (25–29.9 kg/m^2^), and obesity (≥ 30 kg/m^2^) [22].

The HUNT4 self-reported PA questions, assessing frequency, duration, and intensity, were evaluated [17]. The PA questions on frequency, duration and intensity used in HUNT have previously been validated against directly measured maximal oxygen consumption (VO₂max) [23]. Further, the physical activity summary (PAS) score was constructed by multiplying each individual’s response to the three questions as presented in a previous publication [24]. PAS is found to be valid and reliable as published elsewhere [25]. The PA questions used in HUNT4, along with the assigned scores, are presented in Table 1. Individuals who reported ‘never’ or ‘less than once a week’ were assigned a score of ‘zero’ and were regarded as ‘inactive’. The remaining participants were divided into tertiles based on their scores. The inactive group was merged with the low PA group, where “inactive and low PA” had a score of 0-1.5, “moderate PA” had a score of 1.9–3.8, and “high PA” had a score of 3.8–15.

**Table 1:** Physical activity questionnaire at HUNT4 and assigned scores

Question	Response	Response score
**Physical Activity Frequency** “How often do you exercise?”	“Never”	0
	“Less than once a week”	0
	“Once a week”	1
	“Two to three times per week”	2.5
	“Nearly every day”	5
**Physical Activity Intensity** “If you do such exercise as frequently as once or more times a week, how hard do you push yourself?”	“I take it easy; I don’t get out of breath or break a sweat”	1
	“I push myself until I’m out of breath and break into sweat”	2
	“I practically exhaust myself”	3
**Physical Activity Duration** “How long does each session last?”	“Less than 15 min”	0.1
	“15–29 min”	0.38
	“30 min to 1 h”	0.75
	“More than 1 hour”	1.0

To examine the combined effect of BMI and PA on periodontitis stages, four groups were constructed: normal weight and physically active (BMI: 18.5–24.9 kg/m^2^, PAS: 1.9–15), normal weight and physically inactive (BMI: 18.5–24.9 kg/m^2^, PAS: 0-1.5), with overweight-obesity and physically active (BMI: ≥25 kg/m^2^, PAS: 1.9–15), and with overweight-obesity and physically inactive (BMI: ≥25 kg/m^2^, PAS: 0-1.5).

### Covariates

Self-reported individual information on demographics, lifestyle factors, dietary habits, and clinical factors was collected at HUNT4 [17, 26]. Selected confounders included in the analyses were categorized into the following categories: Age was categorized as 20–49, 50–64, and ≥ 65 years after testing for non-linearity in its association with the outcome, as modeling it continuously may influence the results, sex (male, female), household income before taxation was initially collected in Norwegian Kroner and was later converted into euros (< 40,000, 40,000–70,000, > 70,000 euro/year) as presented elsewhere [27]. Smoking status categories were categorized as never, current and former smokers, fruit and vegetable intake variables were merged and categorized into (< 1, 1–3 times, 4 times or more per week). For alcohol intake, participants were asked, “About how often in the last 12 months did you drink alcohol?”. A newly constructed variable for alcohol intake was created and categorized as never drunk/former, monthly, weekly. Diabetes was self-reported and categorized as 'yes' or 'no'. For education, participants were asked, “What is the highest level of education you have completed?”. Originally grouped into six categories in the HUNT 4 questionnaires, education categories were re-grouped into primary school, high school, vocational, university qualifying or more. Missing responses in the questionnaires were reported as “missing category”.

### Statistical analyses

The statistical analyses were conducted using STATA 18.0. Descriptive statistics were presented as frequency (percentage) for categorical variables. The characteristics of the study population were presented by BMI categories as well as combinations of BMI and PA levels. We assessed the association between BMI categories (normal [reference group], overweight, and obesity), PA (inactive-low [reference group], moderate, and high), and periodontitis using modified Poisson regression models with robust error variance. To examine the combined effect of BMI and PA on periodontitis stages, we analyzed the four defined groups: normal weight and physically active (as the reference group), normal weight and physically inactive, with overweight-obesity and physically active, and with overweight-obesity and physically inactive. The outcome variable, periodontitis, was classified into four stages (I, II, III, IV). Additionally, in our analysis, stage 0 was defined as no evidence of periodontitis.

For analysis, the stages were dichotomized as follows:

- Healthy/non-severe (coded as 0): Stage 0, I, and II
- Severe Periodontitis (coded as 1): Stage III and IV

Prevalence Ratios (PR) and corresponding 95% confidence intervals were computed to evaluate the associations. Two models were constructed. Model 1 was unadjusted, and Model 2 was adjusted for potential confounders, including age, sex, smoking, fruit and vegetable intake, alcohol intake, education level, income, and self-reported diabetes status. Consideration of these confounders was based on prior knowledge [28–32]. Furthermore, we assessed effect modification by sex (males and females) and age (< 65 years and ≥ 65 years) using the likelihood ratio test (LRT). To assess the robustness of the findings, sensitivity analyses were conducted. Specifically, BMI and PA were mutually adjusted in their respective models. Additional sensitivity analyses were conducted to examine whether combining participants with overweight and obesity influenced the observed associations, acknowledging that BMI does not distinguish between fat mass and lean mass and may therefore led to some degree of misclassification, particularly among physically active individuals. In these analyses, BMI was categorized as normal weight, overweight, or obesity and jointly combined with physical activity status (physically active vs. physically inactive) to create six BMI–physical activity exposure groups. These groups were evaluated in relation to severe periodontitis (Stage III–IV) using the same multivariable-adjusted models as in the primary analyses.

### Ethical considerations

Informed consent was obtained from all the participants. The current study was performed in accordance with relevant guidelines and regulations and was evaluated and approved by the Norwegian Regional Committees for Medical and Health Research Ethics (REK: 138564).

## Results

A total of 4,384 individuals were included in the study population, of which 54.1% were female participants. The normal-weight group had a higher proportion of younger adults (19–49 years) compared to the overweight and obesity groups. Individuals with obesity who were physically inactive were more likely to have less than university-level education, be current smokers, and have an income below 40,000 euro/year. Severe periodontitis (Stage III/IV) was more prevalent in the group with obesity (Tables 2 and 3).

**Table 2:** Characteristics of participants according to different categories of body mass index (BMI) in the HUNT4 oral health study

Variable	Body Mass Index (BMI, Kg/m^2^) *N* = 4,485		
	Normal *N* = 1,**594** *n* (%)	With overweight *N* = 1,**875** *n* (%)	With obesity *N* = 1,**016** *n* (%)
Sex			
Female	1,017 (63.8)	914 (48.8)	563 (55.4)
Male	577 (36.2)	961 (51.3)	453 (44.6)
Age, years			
20–49	900 (56.5)	759 (40.5)	443 (43.6)
50–64	390 (24.5)	660 (35.2)	359 (35.3)
65 and above	304 (19.1)	456 (24.3)	214 (21.1)
Education			
Primary school	81 (5.4)	138 (7.3)	72 (7.1)
High School	419 (26.3)	509 (27.2)	291 (28.6)
Vocational	270 (16.9)	408 (21.8)	236 (23.23)
University level or more	817 (51.3)	813 (43.4)	406 (39.9)
Income, euro/year			
< 40,000	413 (25.9)	426 (22.7)	280 (27.6)
40,000–70,000	402 (25.2)	564 (30.1)	299 (29.4)
> 70,000	745 (46.7)	845 (45.1)	421 (41.4)
Smoking Status			
Never	835 (52.4)	806 (42.9)	411 (40.5)
Former	626 (39.3)	918 (48.9)	510 (50.2)
Current	126 (7.9)	144 (7.7)	88 (8.7)
Fruit and vegetable intake, per week			
< 1 time	20 (1.3)	27 (1.4)	9 (0.9)
1–3 times	290 (18.2)	375 (20.0)	212 (20.9)
4 times or more	1,282 (80.4)	1,468 (78.3)	789 (77.7)
Alcohol Intake			
Never drunk/former	145 (9.1)	133 (7.1)	82 (8.1)
Monthly	1,108 (69.5)	1,282 (68.4)	741 (7.9)
Weekly	329 (20.6)	444 (23.7)	178 (17.5)
Self-reported Diabetes			
No	1,557 (97.7)	1,778 (94.8)	905 (89.1)
Yes	22 (1.4)	81 (4.3)	93 (9.2)
Periodontitis stages			
no Periodontitis/Stage I	807 (50.6)	716 (38.2)	421 (41.4)
Stage II	580 (36.4)	821 (43.8)	401 (39.5)
Stage III	183 (11.5)	296 (15.8)	167 (16.4)
Stage IV	24 (1.5)	42 (2.2)	27 (2.7)
Physical Activity Levels (PAS Score)			
Inactive/low	492 (30.9)	750 (40.0)	462 (45.5)
Moderate	525 (32.9)	586 (31.3)	336 (33.1)
High	543 (34.1)	503 (26.8)	187 (18.4)

BMI variable was categorized according to the WHO guidelines: normal weight (18.5–24.9 kg/m2), overweight (25–29.9 kg/m2), and obesity (≥ 30 kg/m2); periodontitis stages categorized according to the 2017 classification of periodontal and Peri-Implant diseases and Conditions; physical activity summary score (PAS) categorization: Inactive/Low PA: PAS = 0-1.5; moderate PA: PAS = 1.9–3.8; high PA: PAS = 3.8–15

**Table 3:** Characteristics of participants according to leisure time physical activity levels in the HUNT4 oral health study

Variable	Physical Activity Summary Score (PAS) *N* = 4,384		
	Inactive/low *N* = 1,**704** *n* (%)	Moderate *N* = 1,**447** *n* (%)	High *N* = 1,**233** *n* (%)
Sex			
Female	902 (52.9)	876 (60.5)	649 (52.6)
Male	802 (47.1)	571 (39.5)	584 (47.4)
Age, years			
20–49	841 (49.4)	667 (46.1)	533 (44.9)
50–64	472 (27.7)	503 (34.8)	411 (33.3)
65 and above	391 (22.9)	277 (19.1)	269 (21.8)
Education			
Primary school	152 (8.9)	66 (4.6)	63 (5.1)
High School	490 (28.8)	381 (26.3)	319 (25.9)
Vocational	384 (22.5)	280 (19.4)	233 (18.9)
University qualifying or more	672 (39.4)	716 (49.5)	617 (50.0)
Income, euro/year			
< 40,000	486 (28.5)	329 (22.7)	269 (21.8)
40,000–70,000	510 (29.9)	394 (27.2)	331 (26.9)
> 70,000	672 (39.4)	701 (48.5)	613 (49.7)
Smoking status			
Never	697 (40.9)	691 (47.7)	628 (50.9)
Former	790 (46.4)	673 (46.5)	538 (43.6)
Current	212 (12.4)	78 (5.4)	65 (5.3)
Fruit and vegetable intake, per week			
< 1 time	37 (2.2)	13 (0.9)	6 (0.5)
1–3 times	423 (24.8)	242 (16.7)	194 (15.7)
4 times or more	1,241 (72.8)	1,191 (82.3)	1,033 (83.8)
Alcohol Intake			
Never drunk/former	167 (9.8)	103 (7.1)	77 (6.2)
Monthly	1.231 (72.2)	1,009 (69.7)	846 (68.6)
Weekly	302 (17.7)	329 (22.7)	309 (25.1)
Self-reported Diabetes			
No	1,590 (93.3)	1,376 (95.1)	1,182 (95.9)
Yes	97 (5.7)	51 (3.5)	44 (3.6)
Periodontitis stages			
Not periodontitis/Stage I	743 (43.6)	635 (43.9)	528 (42.8)
Stage II	643 (37.7)	612 (42.3)	514 (41.7)
Stage III	274 (16.1)	176 (12.2)	171 (13.9)
Stage IV	44 (2.6)	24 (1.7)	20 (1.6)
BMI levels			
Normal weight	492 (28.9)	525 (36.3)	543 (44.0)
Overweight	750 (44.0)	586 (40.5)	503 (40.8)
Obesity	462 (27.1)	336 (23.2)	187 (15.2)

Physical activity summary score (PAS) categorization: Inactive/Low PA: PAS = 0-1.5; moderate PA: PAS = 1.9–3.8; high PA: PAS = 3.8–15; BMI variable was categorized according to the WHO classification: normal weight (18.5–24.9 kg/m2), overweight (25–29.9 kg/m2), and obesity (≥ 30 kg/m2)

Table 4 presents the adjusted PRs and 95% CIs for severe periodontitis in relation to BMI and PA levels. The prevalence of severe periodontitis was 19% higher among individuals with obesity compared to those with normal weight (adjusted PR _with obesity_,1.19, 95% CI 1.01, 1.39). Conversely, individuals engaging in moderate PA had a 14% lower prevalence of severe periodontitis compared to those with no or low levels of PA (adjusted PR _moderate_ 0.86, 95% CI 0.74, 0.99).

**Table 4:** PRs and 95% CIs for severe periodontitis (Stage III-IV) in relation to BMI and PA levels

BMI (Kg/m2)	*N* = 4,485	Model 1 Unadjusted PRs (95%CI)	Model 2 Adjusted PRs (95%CI)
Normal	1,594	1 (Reference)	1 (Reference)
Overweight	1,875	1.39 (1.18, 1.63)	1.09 (0.95, 1.26)
Obesity	1,016	1.47 (1.23, 1.76)	1.19 (1.01, 1.39)

PAS	*N* = 4,384		
Inactive-Low	1,707	1 (Reference)	1 (Reference)
Moderate	1,447	0.74 (0.63, 0.87)	0.86 (0.74, 0.99)
High	1,233	0.83 (0.70, 0.98)	0.95 (0.82, 1.11)

*PR* Prevalence ratio, *BMI* Body mass index, PAS: Physical activity summary score Inactive/Low PA: PAS = 0-1.5; Moderate PA: PAS = 1.9–3.8; High PA: PAS = 3.8–15; Model 1: Unadjusted; Model 2: age (20–49, 50–64, 65 and above years), sex (male, female), smoking (Never, former, current), fruit and vegetable intake (< 1, 1–3 times, 4 times or more per week), alcohol (Never drunk/former, monthly, weekly), education (primary school, high school, vocational, university qualifying or more), and income (< 40,000, 40,000–70,000, > 70,000 euro/year) and diabetes status (yes, no)

Table 5 presents the adjusted PRs and 95% CIs for the combined association of BMI and PA. The prevalence of severe periodontitis was 23% higher in individuals who were with overweight-obesity and physically inactive than in individuals who were normal weight and physically active (adjusted PR _with overweight−obesity, inactive_ 1.23, 95% CI 1.02, 1.49).

**Table 5:** PRs and 95% CIs for severe periodontitis (Stage III-IV) in relation to the different combined associations of PA and BMI

Combined BMI, PA	*N* = 4,384	Adjusted Model PRs (95%CI)
Normal weight, physically active	1,068	1 (Reference)
With overweight-obesity, physically active	1,612	1.15 (0.95, 1.38)
Normal weight, physically inactive	492	1.14 (0.89, 1.47)
With overweight-obesity, physically inactive	1,212	1.23 (1.02, 1.49)

*PR* Prevalence ratio, *BMI* Body mass index, *PA* Physical activity

Adjusted Model: age (20–49, 50–64, 65 and above years), sex (male, female), smoking (Never, former, current), fruit and vegetable intake (< 1, 1–3 times, 4 times or more per week), alcohol (Never drunk/former, monthly, weekly), education (primary school, high school, vocational, university qualifying or more), and income (< 40,000, 40,000–70,000, > 70,000 euro/year) and diabetes status (yes, no); normal weight and physically active (BMI: 18.5–24.9 kg/m^2^, Physical activity summary score (PAS): 1.9–15), normal weight and physically inactive (BMI: 18.5–24.9 kg/m^2^, PAS: 0-1.5), with overweight-obesity and physically active (BMI: ≥25 kg/m^2^, PAS: 1.9–15), and with overweight-obesity and physically inactive (BMI: ≥25 kg/m^2^, PAS: 0-1.5)

Furthermore, subgroup analyses revealed no statistical evidence of effect modification by sex (See Additional File 1) or by age (< 65 vs. ≥65 years) (See Additional File 2).

In the sensitivity analyses, mutual adjustment for BMI in PA models and vice versa, obesity remained associated with a higher prevalence of severe periodontitis (adjusted PR _with obesity_ 1.20, 95% CI 1.01, 1.42). However, the inverse association between moderate PA and severe periodontitis was slightly attenuated (adjusted PR _moderate_ 0.87, 95% CI 0.74, 1.01). (See Additional Files 3 and 4). Further, compared with physically active individuals of normal weight, physically inactive individuals with obesity had a higher prevalence of severe periodontitis (adjusted PR _with obesity, inactive_ 1.32; 95% CI: 1.05, 1.67), whereas this association was attenuated among physically active individuals with obesity (adjusted PR _with obesity, active_ 1.21; 95% CI: 0.96, 1.53). These sensitivity analyses yielded results consistent with the main analyses (see additional File 5).

## Discussion

In this population-based cross-sectional study of 4,384 adults, being with obesity (≥ 30 Kg/m^2^) was associated with a higher prevalence of severe periodontitis, while moderate PA was associated with a lower prevalence of severe periodontitis after adjustments for potential confounders. Moreover, physically inactive individuals with overweight or obesity had a higher prevalence of severe periodontitis. These findings support our hypotheses that low PA and excess body weight exert a combined effect on the prevalence of severe periodontitis. Furthermore, there was no effect modification observed by age or by sex.

This study has several strengths. First, it includes both male and female adult participants who have undergone a full mouth periodontal examination and assessment based on the updated EFP/AAP 2018 classification [1, 19]. This represents a notable improvement over previous studies, which often relied on non-validated methods, self-reported data, or outdated classifications of periodontitis. The EFP/AAP 2018 classification represents the most current standard, offering several strengths, including the introduction of periodontitis staging and grading. This enables dentists to develop individualized diagnostic and treatment plans for each patient [3]. Second, an extensive range of potential confounders was included in the regression models. Third, weight and height were measured by trained professionals [17], rather than being self-reported. Lastly, the PAS in the HUNT study was constructed using PA frequency, duration, and intensity of activity. The PAS was externally validated and is considered a reliable measure [25].

However, this study is not without limitations. There is a potential for social desirability bias or recall bias due to participants’ self-reported HUNT4 questionnaires on PA measures, including frequency, duration, and intensity, as well as other lifestyle factors used as covariates, such as smoking and alcohol consumption. Furthermore, the study sample was drawn from a relatively small and homogeneous population in the Northern region of Trøndelag county, which may limit the generalizability of the findings to broader or more diverse populations. The participation rate in HUNT4 was 54%, which is lower than in previous HUNT surveys. Differences between participants and non-participants have been documented, indicating that selection bias may have occurred at the time of inclusion into HUNT4 and again in the sub-study, HUNT4 Oral Health Study [26]. Consequently, the generalizability of the present findings to the general population may be limited to a broader population. Although BMI does not distinguish between fat mass and lean mass and may therefore result in some degree of misclassification, particularly among physically active individuals, our sensitivity analyses separating individuals with obesity from those with overweight showed similar patterns of association. This suggests that such misclassification is unlikely to have substantially influenced the main findings. Nevertheless, future studies using direct measures of body composition (e.g., dual-energy X-ray absorptiometry or bioelectrical impedance analysis) would be valuable to further clarify these associations. Finally, we cannot rule out the issue of residual confounding. For example, variations in smoking exposure, such as smoking pack-years, were not accounted for, and data on the presence of plaque, an important risk factor for periodontitis, were omitted in clinical examinations in the HUNT4 Oral Health Study.

Consistent with our findings, a 2010 systematic review and meta-analysis, primarily based on cross-sectional studies, and a more recent 2022 review, which included 29 cross-sectional, 4 cohort, and 4 case-control studies, have reported a positive association between obesity and periodontitis [33, 34]. However, a cross-sectional study from Denmark of adults aged≥20 (*N* = 2,951) reported an inverse association between obesity and clinical attachment loss (CAL), but a positive association with BoP [35]. These divergent findings may be attributed to the study’s smaller sample size and its limited adherence to the 2018 EFP/AAP classification criteria. Another cross-sectional study from Australia (*N* = 4,170) of individuals aged 15 years or older reported no association between overweight/obesity and periodontitis. Notably, this study applied the Centers for Disease Control and Prevention (CDC) and American Academy of Periodontology (AAP) case definition of periodontitis, but did not adjust for other important confounders, only for age, sex, smoking, and reason for dental visits [36]. Both studies relied on self-reported BMI [35, 36], which may have also introduced measurement bias, unlike our study.

Consistent with our study findings, previous cross-sectional studies have reported an inverse association between PA and periodontitis [37–39]. Al-Zahrani et al. reported that the PA was associated with a lower periodontitis prevalence (OR 0.58, 95% CI 0.35, 0.96), after adjusting for confounders [37]. A recent systematic review including 14 observational studies found that physically active individuals exhibited lower levels of inflammatory biomarkers, which were linked to improved periodontal health [40]. Similarly, another recent systematic review and meta-analysis (one case-control, one cohort, and five cross-sectional studies) published in 2020, suggested that physically active individuals were 22% less likely to have periodontal disease compared to their inactive counterparts (OR 0.78 95% CI 0.65, 0.93) [11]. Nevertheless, other studies report mixed findings. In a cross-sectional study by Marruganti et al., leisure time PA was associated with a lower risk of periodontitis (OR 0.81, 95% CI 0.72, 0.92), while occupational PA was associated with a higher risk of periodontitis (OR 1.16, 95% CI 1.04, 1.30) [12]. In a study by Baumeister et al., where they used genetic variants as instrumental variables, no evidence of associations between PA on periodontitis risk was found [13]. This inconsistency may be attributed to differences in study design, definitions of periodontitis, types of PA (leisure time or occupational PA), and methodologies used.

Further, prior research examining the combined association of BMI and PA with severe periodontitis is limited. Consistent with our findings, a study of 1160 Japanese individuals reported that physical fitness and weight may exert a combined effect on periodontal health, and those with the lowest BMI and highest VO2 max showed lower odds of severe periodontitis (OR 0.17, 95% CI 0.05, 0.55) [41]. However, that study assessed periodontal conditions using the Community Periodontal Index (CPI), evaluated obesity through BMI and percentage of body fat, and measured fitness via maximal oxygen consumption (VO2 max). These methods, however, may differ from the more recent periodontitis classification and could vary in reliability.

Age and sex are well-known risk factors for periodontitis [32]. In the present study, although the prevalence of severe periodontitis varies slightly by age and sex, the magnitude of the association between BMI, PA, and periodontitis does not appear to differ significantly across these groups in this population, suggesting no effect modification by age and sex.

## Conclusion

This cross-sectional study examined the associations between body mass index, physical activity, and severe periodontitis, revealing that obesity was associated with a higher prevalence of severe periodontitis, whereas moderate physical activity was associated with a lower prevalence. Notably, the highest prevalence of severe periodontitis was observed among individuals with overweight or obesity who were physically inactive, suggesting a potential combined effect of excess body weight and physical inactivity on periodontal health. Although the cross-sectional design precludes the establishment of temporal or causal relationships, the findings highlight a potential protective role of physical activity in mitigating the adverse impact of obesity on periodontitis severity. Future longitudinal studies using the 2018 EFP/AAP periodontitis classification are needed to validate and further clarify these associations and should incorporate direct measures of body composition to overcome the limitations of BMI.

## Supplementary Information

Supplementary Material 1.

## Data Availability

Due to restrictions imposed by the HUNT Research Centre, in accordance with the Norwegian Data Inspectorate, data cannot be made publicly available. The personal identification number provided to all Norwegians at birth or immigration serves as the primary means of identification in the database, and de-identified data are sent to researchers after a research protocol has been approved by the Regional Ethical Committee and HUNT Research Centre. HUNT Research Centre cannot deposit data in open repositories and attempts to limit storage of data outside HUNT databank in order to safeguard participants’ privacy. All data transmitted to other projects are precisely documented in the HUNT databank, which is able to reproduce them upon request to the HUNT Data Access Committee (kontakt@hunt.ntnu.no). Please visit (https://www.ntnu.edu/hunt/data) for more details.

## Abbreviations

BMI: Body Mass Index
PA: Physical Activity
STROBE: STrengthening the Reporting of OBservational studies in Epidemiology
HUNT: The Trøndelag Health Study
EFP/AAP 2018 classification: The Classification of Periodontal and Peri-Implant Diseases and Conditions
PPD: Periodontal probing depths
BoP: Bleeding on probing
BW: Bitewing
OPG: Orthopantomogram radiographs
CEJ: The cementoenamel junction
AC: The alveolar bone crest
RBL: The radiographic bone loss
WHO: World Health Organization
PAS: Physical activity summary score
PR: Prevalence Ratios
REK: The Norwegian Regional Committees for Medical and Health Research Ethics
CAL: Clinical attachment loss
CDC: The Centers for Disease Control and Prevention
AAP: American Academy of Periodontology
CPI: Community Periodontal Index
VO2 max: Maximal oxygen consumption
