# Supplementary material for: Body mass index, physical activity, and severe periodontitis– a cross-sectional HUNT4 Oral Health Study
Source: BMC Oral Health. 2026 Feb 18;26:404. doi: 10.1186/s12903-026-07882-x (PMC12952053; doi:10.1186/s12903-026-07882-x)
Supplement: Supplementary file 1 — Supplementary Material 1. [file 12903_2026_7882_MOESM1_ESM.docx]

**Additional Files**

1. **Subgroup Analysis**

**Additional File 1. Cross-sectional association between body mass index, physical activity, its combination with physical activity and periodontitis in HUNT4 Oral Health Study stratified by sex**

| **BMI (Kg/m^2^)** | **N**  **1,991** | **Males**  **Adjusted Model**  **PRs (95%CI)** | **N**  **2, 494** | **Females**  **Adjusted Model**  **PRs (95%CI)** | **LRT**  ***p-*value** |
| --- | --- | --- | --- | --- | --- |
| Normal |  | 1 (Reference) |  | 1 (Reference) | 1.85 |
| Overweight |  | 1.09 (0.87, 1.38) |  | 1.06 (0.88, 1.28) |  |
| Obesity |  | 1.39 (1.09, 1.80) |  | 1.05 (0.83, 1.31) |  |
| **PAS** | **N**  **1,957** |  | **N**  **2,427** |  |  |
| Inactive-Low |  | 1 (Reference) |  | 1 (Reference) | 0.91 |
| Moderate |  | 0.87 (0.69, 1.10) |  | 0.85 (0.69, 1.04) |  |
| High |  | 1.01 (0.81, 1.25) |  | 0.89 (0.72, 1.12) |  |
| **BMI, PA** | **N 1,957** |  | **N**  **2,427** |  |  |
| Normal weight, physically active |  | 1 (Reference) |  | 1 (Reference) | 1.04 |
| With overweight-obesity, physically active |  | 1.21 (0.90, 1.63) |  | 1.08 (0.85, 1.38) |  |
| Normal weight, physically inactive |  | 1.11 (0.74, 1.66) |  | 1.18 (0.86, 1.62) |  |
| With overweight-obesity, physically inactive |  | 1.26 (0.93, 1.71) |  | 1.21 (0.95, 1.55) |  |

LRT: Likelihood ratio test; PR: Prevalence ratio; BMI: Body mass index; PAS: Physical activity summary score Inactive/Low PA: PAS = 0-1.5; Moderate PA: PAS = 1.9-3.8; High PA: PAS = 3.8-15; Adjusted Model: age (20-49, 50-64, 65 and above years), sex (male, female), smoking (Never, former, current), fruit and vegetable intake (<1, 1-3 times, 4 times or more per week), alcohol (Never drunk/former, monthly, weekly), education (primary school, high school, vocational, university qualifying or more), and income (<40,000, 40,000-70,000, >70,000 euro/year) and diabetes status (yes, no); normal weight and physically active (BMI: 18.5-24.9 kg/m^2^ , PAS: 1.9-15), normal weight and physically inactive (BMI: 18.5-24.9 kg/m^2^, PAS: 0-1.5), With overweight-obesity and physically active (BMI: ≥25 kg/m^2^, PAS: 1.9-15), and with overweight-obesity and physically inactive (BMI: ≥25 kg/m^2^, PAS: 0-1.5)

**Additional File 2. Cross-sectional association between body mass index, physical activity, its combination with physical activity and periodontitis in HUNT4 Oral Health Study stratified by age**

| **BMI (Kg/m2)** | **N**  **3,511** | **<65**  **Adjusted Model**  **PRs (95%CI)** | **N**  **974** | ≥ **65**  **Adjusted Model**  **PRs (95%CI)** | **LRT**  ***p-*value** |
| --- | --- | --- | --- | --- | --- |
| Normal |  | 1 (Reference) |  | 1 (Reference) | 1.55 |
| Overweight |  | 1.09 (0.86, 1.38) |  | 1.08 (0.91, 1.29) |  |
| Obesity |  | 1.32 (1.03, 1.70) |  | 1.08 (0.86, 1.32) |  |
| **PAS** | **N**  **3,447** |  | **N**  **937** |  |  |
| Inactive-Low |  | 1 (Reference) |  | 1 (Reference) | 1.09 |
| Moderate |  | 0.81 (0.63, 1.02) |  | 0.89 (0.73, 1.07) |  |
| High |  | 0.97 (0.76, 1.24) |  | 0.91 (0.75, 1.11) |  |
| **Joint BMI, PA** | **N**  **3,447** |  | **N**  **937** |  |  |
| Normal weight, physically active |  | 1 (Reference) |  | 1 (Reference) | 0.88 |
| With overweight-obesity, physically active |  | 1.18 (0.88, 1.57) |  | 1.13 (0.89, 1.43) |  |
| Normal, physically inactive |  | 1.15 (0.78, 1.71) |  | 1.20 (0.88, 1.64) |  |
| With overweight-obesity, physically inactive |  | 1.32 (0.97, 1.78) |  | 1.21 (0.95, 1.53) |  |

LRT: likelihood ratio test; PR: Prevalence ratio; BMI: Body mass index; PAS: Physical activity summary score Inactive/Low PA: PAS = 0-1.5; Moderate PA: PAS = 1.9-3.8; High PA: PAS = 3.8-15; Adjusted Model: age (20-49, 50-64, 65 and above years), sex (male, female), smoking (Never, former, current), fruit and vegetable intake (<1, 1-3 times, 4 times or more per week), alcohol (Never drunk/former, monthly, weekly), education (primary school, high school, vocational, university qualifying or more), and income (<40,000, 40,000-70,000, >70,000 euro/year) and diabetes status (yes, no); normal weight and physically active (BMI: 18.5-24.9 kg/m^2^ , PAS: 1.9-15), normal weight and physically inactive (BMI: 18.5-24.9 kg/m^2^, PAS: 0-1.5), With overweight-obesity and physically active (BMI: ≥25 kg/m^2^, PAS: 1.9-15), and with overweight/obesity and physically inactive (BMI: ≥25 kg/m^2^, PAS: 0-1.5)

**Sensitivity Analyses**

| **BMI (Kg/m2)** | **N=4, 384** | **Adjusted Model**  **PRs (95%CI)** |
| --- | --- | --- |
| Normal |  | 1 (Reference) |
| Overweight |  | 1.09 (0.94, 1.26) |
| Obesity |  | 1.20 (1.01, 1.42) |

**Additional File 3. PRs and 95% CIs for severe periodontitis (Stage III-IV) in relation to BMI**

PR: Prevalence ratio; BMI: Body mass index; Adjusted Model: physical activity levels (PAS); age (20-49, 50-64, 65 and above years), sex (male, female), smoking (Never, former, current), fruit and vegetable intake (<1, 1-3 times, 4 times or more per week), alcohol (Never drunk/former, monthly, weekly), education (primary school, high school, vocational, university qualifying or more), income (<40,000, 40,000-70,000, >70,000 euro/year) and diabetes status (yes, no), Poisson regression analysis

| **PAS** | **N=4,384** | **Adjusted Model**  **PRs (95%CI)** |
| --- | --- | --- |
| Inactive-Low |  | 1 (Reference) |
| Moderate |  | 0.87 (0.74, 1.01) |
| High |  | 0.98 (0.84, 1.15) |

**Additional File 4. PRs and 95% CIs for severe periodontitis (Stage III-IV) in relation to PA levels**

PR: Prevalence ratio; PAS: Physical activity summary score Inactive/Low PA: PAS = 0-1.5; Moderate PA: PAS = 1.9-3.8; High PA: PAS = 3.8-15; Adjusted Model: BMI (continuous), age (20-49, 50-64, 65 and above years), sex (male, female), smoking (Never, former, current), fruit and vegetable intake (<1, 1-3 times, 4 times or more per week), alcohol (Never drunk/former, monthly, weekly), education (primary school, high school, vocational, university qualifying or more), and income (<40,000, 40,000-70,000, >70,000 euro/year) and diabetes status (yes, no), Poisson regression analysis

**Additional File 5.** **PRs and 95% CIs for severe periodontitis (Stage III-IV) in relation to the different combined associations of PA and BMI**

| **Combined BMI, PA** | **N=4,384** | **Adjusted Model**  **PRs (95%CI)** |
| --- | --- | --- |
| Normal weight, physically active  Normal weight, physically inactive  With Overweight, physically active  With Overweight, physically inactive  With obesity, physically active  With obesity, physically inactive | 1,068 492 1,089 750 523 462 | 1 (Reference)  1.14 (0.89, 1.47) 1.12 (0.92, 1.36) 1.19 (0.97, 1.45) 1.21 (0.96, 1.53) 1.32 (1.05, 1.67) |

PR: Prevalence ratio; BMI: Body mass index; PA: Physical activity; Adjusted Model: age (20-49, 50-64, 65 and above years), sex (male, female), smoking (Never, former, current), fruit and vegetable intake (<1, 1-3 times, 4 times or more per week), alcohol (Never drunk/former, monthly, weekly), education (primary school, high school, vocational, university qualifying or more), and income (<40,000, 40,000-70,000, >70,000 euro/year) and diabetes status (yes, no); normal weight, physically active (BMI: 18.5-24.9 kg/m^2^ , Physical activity summary score (PAS): 1.9-15), normal weight, physically inactive (BMI: 18.5-24.9 kg/m^2^ , PAS: 0-1.5), with overweight, physically active (BMI: 25.0-29.9 kg/m^2^ , PAS: 1.9-15), with overweight, physically inactive (BMI: 25.0-29.9 kg/m^2^ , PAS: 0-1.5), with obesity, physically active (BMI: ≥ 30.0 kg/m^2^ , PAS: 1.9-15), with obesity, physically inactive (BMI: ≥ 30.0 kg/m^2^ , PAS: 0-1.5),
